# Lithotomy and Lithotrity

**Published:** 1847-01

**Authors:** 


*Lithotomy and Lithotrity.*—Until very lately, patients applying to surgeons were constantly recommended to submit to the knife, in order to get rid of stone in the bladder, whatever might be the size of the concretion, or the state of the urinary organs. On the other hand, if he fell into the hands of the professed grinder, no matter what the peculiarities of his case, he was as certain to be subjected to the boring or hammering processes. Now that the merits of both operations are better understood and appreciated—some few surgeons having thought proper to turn their attention to this matter, and study and understand lithotrity as well as lithotomy—patients have a chance of being treated judiciously and conscientiously, and of having that proceeding resorted to which is adapted to their respective cases. I was not slow to adopt the operation of crushing, have always had a favorable impression of it, and have throughout used the same language regarding it, yet I have the credit of being an opponent to lithotrity. I have all along been, and am certainly still, opposed to the abuse of any one operation, by its indiscrminate employment in all cases, and by its being practised by those alone who know no other. It can be employed safely only by those who understand well the healthy anatomy of the urethra and bladder; who are acquainted with their sympathies, vital actions, and pathological changes; and who both understand, and are in the constant habit of treating derangement of their functions. The operation of lithotrity is applicable to patients above the age of puberty, when the symptoms have not endured very long, when the foreign body is ascertained to be about the size of the one sketched two pages back—measuring six or seven lines, or even more, perhaps, say a large as a chestnut;—when the bladder and urethra are in a tolerably healthy and normal condition—as indicated by the power to retain the urine comfortably for several hours, and to pass it in a tolerably free stream; and when the viscus admits of injection and a careful exploration.

The operation of lithotomy must yet continue to be performed on children, and on those of mature age who are so ill-informed or foolish as to permit the stone to attain an inordinate bulk. The concretions in young subjects are generally composed of a very dense substance, the oxalate of lime, in whole or in part; and the urethra is often so narrow as to preclude the application of instruments strong enough to reduce them to fragments. The reason for givihg a preference to incision over crushing, when the stone is large, have already been given.

This favorable opinion of lithotrity was published in 1840, since when it has been confirmed and much strengthened by ample experience. Of late years, in point of fact, I have scarcely been obliged to have recourse to lithotomy at all in private practice. At the hospital, patients yet present themselves with large stones and bad bladders. Then lithotomy is both a less painful and much more safe operation, as already propounded. During the above period twenty-four patients have been cut, and all have recovered without accident; these patients have been of all ages, from two to eighty years of age, and some of them not over favorable subjects. So that, after all, there is not much to find fault with as regards this cruel and bloody 'operation' when carefully set about.

*Liston's Practical Surgery.*

